# A novel large germ line deletion in adenomatous polyposis coli *(APC)* gene associated with familial adenomatous polyposis

**DOI:** 10.1002/mgg3.479

**Published:** 2018-09-26

**Authors:** Farzaneh Pouya, Afsaneh Mojtabanezhad Shariatpanahi, Kamran Ghaffarzadegan, Seyed Abbas Tabatabaee Yazdi, Hamed Golmohammadzadeh, Ghodratollah Soltani, Kian Aminian Toosi, Mohammad Amin Kerachian

**Affiliations:** ^1^ Department of Medical Genetics, Faculty of Medicine Mashhad University of Medical Sciences Mashhad Iran; ^2^ Cancer Genetics Research Unit Reza Radiotherapy and Oncology Center Mashhad Iran; ^3^ Razavi Cancer Research Center, Razavi Hospital Imam Reza International University Mashhad Iran; ^4^ Department of Pathology Mashhad University of Medical Sciences Mashhad Iran; ^5^ Endoscopic and Minimally Invasive Surgery Research Center Mashhad University of Medical Sciences Mashhad Iran; ^6^ Department of Biology Islamic Azad University Mashhad Branch Mashhad Iran; ^7^ Cancer Genetics Research Center Mashhad University of Medical Sciences Mashhad Iran

**Keywords:** *APC* gene, colorectal cancer, Familial adenomatous polyposis, large exon deletion, targeted next‐generation sequencing

## Abstract

**Background:**

Familial adenomatous polyposis (FAP) is a familial colorectal cancer predisposition syndrome characterized by the development of numerous colorectal polyps, which is inherited in an autosomal dominant manner. FAP is caused by germ line mutations in adenomatous polyposis coli (*APC*) gene. Here, we described the identification of a causative *APC* gene deletion associated with FAP in an Iranian family.

**Methods:**

Diagnosis of FAP was based on clinical findings, family history, and medical records (colonoscopy and histopathological data) after the patients were referred to Reza Radiotherapy and Oncology Center, Iran, for colonoscopy. Blood samples were collected, and genomic DNA was extracted. *APC* mutation screening was conducted by target next‐generation sequencing and quantitative real‐time PCR.

**Results:**

A novel heterozygous large deletion mutation, c.(135+1_136–1)_(*2113+1_*2114–1) spanning exon 3 to 16 [EX3_16 DEL] of *APC* gene (GenBank Accession# MG712911), was detected in a proband and all her affected relatives in five generations, which was absent in unaffected family members and normal controls.

**Conclusions:**

This novel deletion is the first report, describing the largest deletion of *APC* gene. Our novel finding contributes to a more comprehensive database of germ line mutations of *APC* gene that could be used in medical practice for the molecular diagnosis, risk assessment susceptibility of the disease for the FAP patients.

## 1 INTRODUCTION

Colorectal cancer (CRC), one of the major causes of morbidity and mortality, accounts for over 9% of all cancer incidences worldwide (Rokni, Shariatpanahi, Sakhinia, & Kerachian, 2018). Between 2% to 5% of all colon cancers arise in the setting of well‐defined inherited syndromes (Jasperson, Tuohy, Neklason, & Burt, 2010). Familial adenomatous polyposis (FAP) [OMIM#175100] is a familial CRC syndrome marked by the development of numerous colorectal adenomas or polyps, which is inherited in an autosomal dominant manner. If not diagnosed and treated, patients often develop CRC by the age of 40–50 years, which accounts for less than 1% of CRC cases (Stoffel et al., 2018; Half, Bercovich, and Rozen, 2009). The incidence of FAP is about 1/8,300 at birth, and it affects both sexes equally. In the majority of FAP patients, not only colorectal adenomas but also various extra‐colonic manifestations were also seen, such as lipomas, osteomas, dental abnormalities, congenital hypertrophy of the retinal pigment epithelium (CHRPE), desmoids tumors, epidermoid cysts, upper gastrointestinal polyps (in the stomach or duodenum), thyroid, brain and hepatobiliary tract cancers (Song, Yuan, Zheng, & Yang, 2013; Zhang et al., 2015).

Based on the number of polyps and the onset age of polyposis and cancer, FAP is classified into two groups: the classical FAP (CFAP) and attenuated FAP (AFAP) (Nagase et al., 1992; Nieuwenhuis & Vasen, 2007). In the CFAP, patients develop hundreds to thousands of colorectal adenomatous, which frequently occur within the second decade and become symptomatic during the third decade of life. If untreated, mean age of CRC patients is about 40 years (Nagase et al., 1992). Patients with AFAP manifest 10 to 100 adenomatous polyps, usually in an older age at diagnosis of both polyposis and CRC than in CFAP (Aretz et al., 2005; Knudsen, Bisgaard, & Bulow, 2003). FAP is caused by germ line mutations in adenomatous polyposis coli (*APC*) gene (Groden et al., 1991; Nishisho et al., 1991). *APC* is often cited as a typical example of a tumor suppressor gene and encodes a large multifunctional protein that plays an important role in the *Wnt*‐signaling pathway. This pathway is involved in variety of cellular processes, including transcription, cell adhesion, cell cycle division, cell migration, and apoptosis (Fearnhead, Britton, & Bodmer, 2001; Vogelstein et al., 1988).

To date, more than thousand different *APC* germ line mutations have been registered in the Human Gene Mutation Database (https://www.hgmd.cf.ac.uk/ac). The majority of *APC* germ line mutations are classified into three main types; splice site mutations, nonsense/frameshift, and large deletions (Nieuwenhuis & Vasen, 2007). In several studies, a direct correlation between the location of *APC* mutations and phenotypic features, including age of onset, number of polyps, and occurrence of extra‐colonic manifestations, has been described. This phenotype–genotype correlation is critical since it would facilitate making the most appropriate prophylactic therapies and surgical procedures (Zhang et al., 2016).

In this study, a novel heterozygous large germ line deletion in *APC* gene in an Iranian FAP family was identified by targeted next‐generation sequencing. Subsequently, quantitative real‐time PCR (qPCR) was performed in order to segregate this germ line mutation in all affected members.

## 2 MATERIALS AND METHODS

### 2.1 Ethical statement

Written informed consent was obtained from each patient involved in this study. The study protocol was reviewed and approved by the Ethical Committee of the Mashhad University of Medical Sciences, Mashhad, Iran. Diagnosis of FAP has been performed by gastroenterologist according to clinical symptoms, endoscopy, and histopathological data after the proband (IV‐11) was referred to Reza Radiotherapy and Oncology Center (RROC), Mashhad, Iran.

### 2.2 Patients and pedigree

Family members of a five Iranian generation with FAP, diagnosed and treated in RROC, were enrolled in this study. The diagnostic standard criteria for patients with FAP were as follows: (a) patients having more than one hundred colorectal adenomas or polyps and (b) at least 20 synchronous adenomatous polyps in patients with a positive family history of FAP. All patients’ clinical information, family history, and the results of colonoscopic and pathologic examinations were collected. Five to ten milliliters of peripheral blood was obtained from as many family members as possible, with full informed consent.

### 2.3 Targeted exome‐based next‐generation sequencing (NGS)

Genomic DNA was extracted from the blood samples utilizing QIAamp DNA blood Mini Kit (Qiagen, Hilden, Germany) according to the manufacturer's protocol.

Extracted DNA of proband (IV‐11) was sequenced using target exome‐based next‐generation sequencing. BGI Clinical Laboratories (Shenzhen, China) enriched and sequenced 14 genes (*APC, AXIN2, EPCAM, MLH1, MLH3, MSH2, MSH6, MUTYH, PMS1, PMS2, STK11, PTEN, SMAD4, and BMPR1A*) related with hereditary CRC by high‐throughput platform. All exons and flanking 10 bp were detected and analyzed. On average, 99.9% of base pairs with >100X coverage were successfully detected. The average of sequencing depth approximated to the sequencing depth median of all exons, which means good randomicity of sequencing.

### 2.4 Quantitative real‐time PCR

In order to validate the results of targeted NGS, as this is a large deletion mutation, quantitative real‐time polymerase chain reaction (qPCR) was performed for exons 3, 9, 12, 14, and 16 of the proband and normal controls in triplicates (primer sequences are available upon request). To segregate the mutation in the family members, the relative DNA copy number for the *APC* exon 14 was quantified by qPCR, in the proband (IV‐11), in all affected family members, in 14 available unaffected family members, and in 3 normal controls with LightCycler® 96 Real‐time PCR System (Roche Life Science, Germany). The relative copy number of *APC* exon 14 was normalized to *APC* exon 1 (a non‐deleted region). Data were analyzed using the comparative threshold cycle (2^–ΔΔ^ *^C^* ^T^) method. The DNA copy number level for the *APC* exon 14 in each sample was compared with the level in control blood samples from three normal individuals. The primers for amplifying *APC* exons were listed in Table 1. The PCR thermal conditions were as follows: an initial denaturation step of 95°C for 15 min, followed by 95°C for 20 s, annealing 58°C for 25 s, and 72°C for 30 s, for a total of 40 cycles. All samples were evaluated by EvaGreen (Solis BioDyne 5x HOT FIREPol® EvaGreen® qPCR Mix Plus, Estonia) qPCR in triplicate, and the mean value was used for quantification. Its efficiency was calculated based on the slope of the standard curve (equation: efficiency = (10^(−1/slope)^ − 1) × 100). Correlation coefficients (*r* ^2^ ≥ 0.99) were considered. Melting curves of all samples were observed carefully to ensure that only one product was amplified. The qPCR assays were performed according to the *MIQE* guidelines.

**Table 1:** Detailed primer sequence for real‐time PCR

Primer	Primer sequence	Size/bp	Tm/°C
Exon 1‐ Forward	5′ ‐ATA GGT CCA AGG GTA GCC‐ 3^′^	190	56.1
Exon 1‐ Reverse	5′ ‐ATA AAC TAC AAT TAA AAG TCA CAG TCT‐ 3′	190	59.2
Exon 14‐ Forward	5′ ‐CTT ACC GGA GCC AGA CAA AC‐ 3′	155	60.5
Exon 14‐ Reverse	5′ ‐GGT CTT TTT GAG AGT ATG AAT TCT G‐ 3′	155	60.9

## 3 RESULTS

### 3.1 Family recruitment and clinical examination

**Figure 1:**
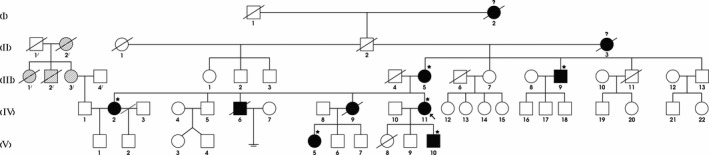
Pedigree structure of the Iranian family with familial adenomatous polyposis. Family members with FAP are indicated with solid shading. Individuals with "question mark" had GI bleeding, and ones with "parallel line shading" had GI malignancy. Squares and circles denoted males and females, respectively. Individuals labeled with a solidus were deceased. Family members detected with APC gene deletion are indicated by with asterisks. Roman numerals indicate generations. Arrow indicates the proband (IV‐11)

**Figure 2:**
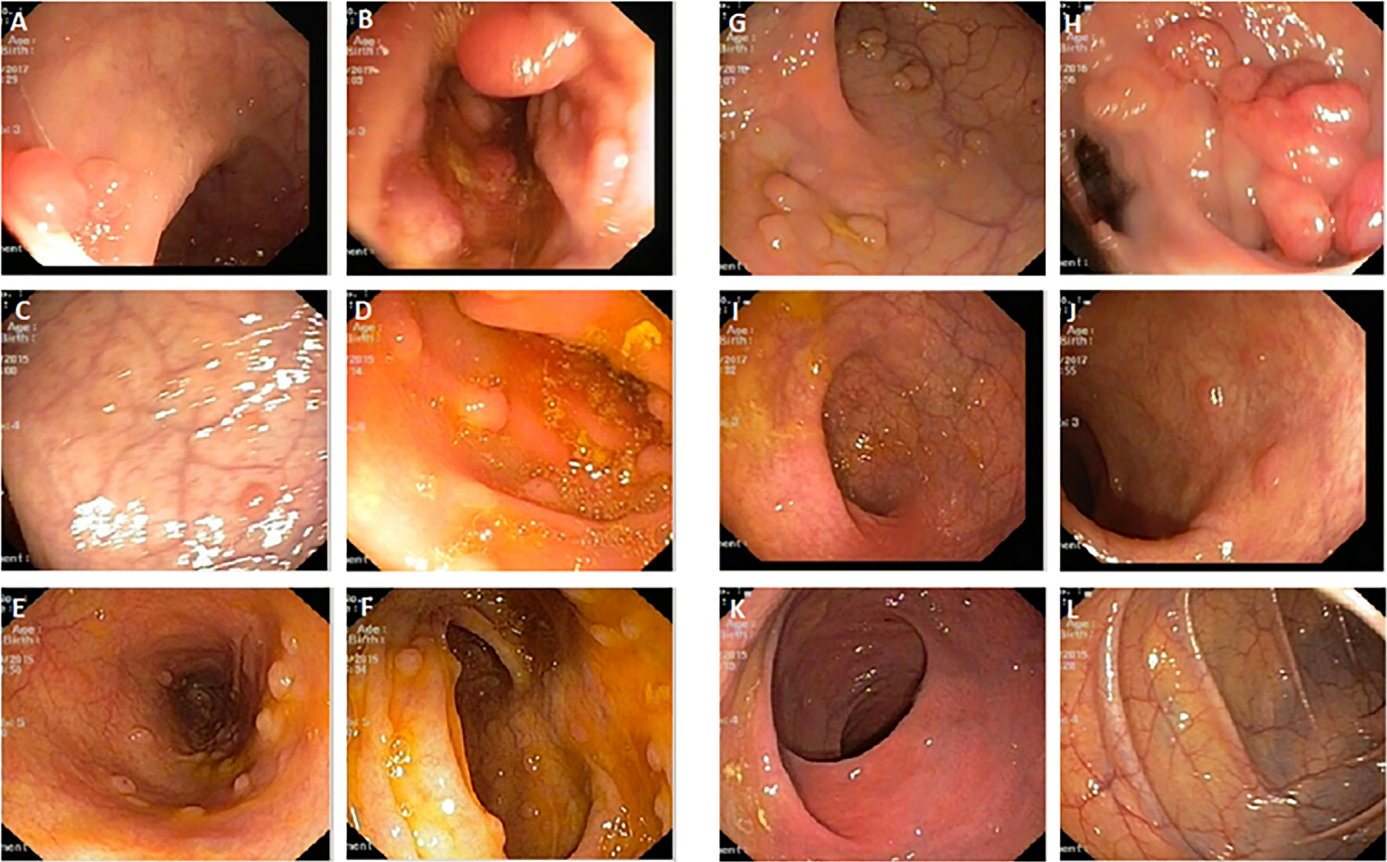
Colonoscopy reports. (a, b) Multiple polyps in the rectum and colon of affected member (III‐5); (c, d) multiple polyps in the rectum and colon of affected member (IV‐2); (e, f) multiple polyps in the rectum and colon of affected member (IV‐11); (g, h) multiple pin the rectum and colon of affected member (V‐5); (i, j) no polyps in the rectum but multiple polyps in the colon of affected member (V‐10); (k, l) no polyps in the rectum and colon of unaffected member (IV‐5)

**Figure 3:**
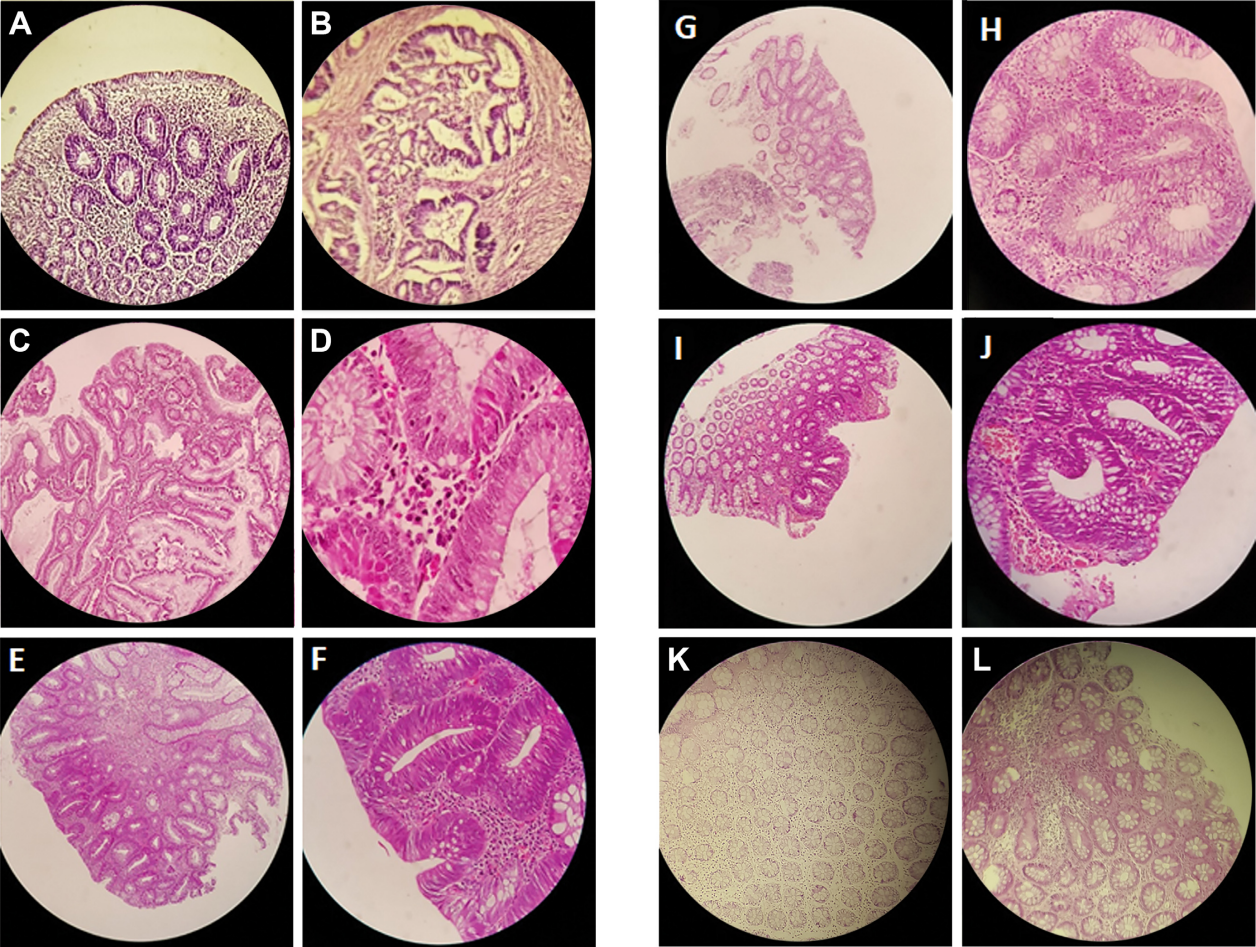
Histopathology results. (a) Small sessile tubular polyp, and (b) an invasive adenocarcinoma in proband (III 5). (c) tubulovillus adenoma and (d) low‐grade dysplasia in proband (III‐9). (e) Tubular adenoma and (f) low‐grade dysplasia in proband (IV‐2). (g) Small sessile tubular poly and H, low‐grade dysplasia in proband (IV‐11). (i) Small sessile tubular adenoma, and J, low‐grade dysplasia in proband (V‐10). (k, l) Unaffected member with normal colonic mucosa

We identified a five‐generation Iranian pedigree with 56 members, which among them eight individuals were affected by FAP including three with CRC (III‐5, IV‐6, IV‐9). Two individuals (IV‐6, IV‐9) had died from CRC. The pedigree suggested autosomal dominant mode of inheritance Figure 1. A detailed and comprehensive clinical information from both affected and unaffected family members is illustrated in Table 2. Colonoscopy and histopathology for all affected family members along with an unaffected member are described in Figures 2 and 3, respectively.

**Table 2:** Clinical characteristics of all the affected and unaffected family members found in our study (bold text: patients)

ID	Family ID	Sex	Exon deletion	Present age	Age at diagnosis	No. of polyps	Pathology result (from surgery)	Clinical manifestation
1	I−1	M	–	Died	–	–		
2	**I−2**	F	–	Died	–	–		
3	II−1	F	–	Died	–	–		
4	II−2	M	–	Died	–	–		
5	**II−3**	F	–	Died	–	–		
6	II−1^/^	M	–	Died	–	–		
7	II−2^/^	F	–	Died	–	–		
8	III−1	F	WT	60	–	–		
9	III−2	M	WT	56	–	–		
10	III−3	M	WT	52	–	–		
11	III−4	M	–	Died	–	–		
12	**III−5**	F	DEL	72	70	>100	Invasive adenocarcinoma	Abdominal pain–constipation–melena–cough–respiratory distress
13	III−6	M	–	Died	–	–		
14	III−7	F	WT	76	–	–	–	
15	III−8	F	–	62	–	–		
16	**III−9**	M	DEL	68	67	>100	Focal high‐grade dysplasia	Asymptomatic
17	III−10	F	–	62	–	–		
18	III−11	M	–	Died	–	–		
19	III−12	F	–	55	–	–		
20	III−13	M	WT	54	–	–		
21	III−1^/^	F	–	Died	–	–		
22	III−2^/^	M	–	Died	–	–		
23	III−3^/^	F	–	60	–	–		
24	III−4^/^	M	–	70	–	–		
25	IV−1	M	–	40	–	–		
26	**IV−2**	F	DEL	33	30	>100	Multiple tubular and tubulovillous adenomas with low‐grade dysplasia	Asymptomatic
27	IV−3	M	–	37	–	–		
28	IV−4	F	–	44	–	–		
29	IV−5	M	WT	54	–	–		
30	**IV−6**	M	–	Died	50	>100	Adenocarcinoma	Hemorrhoid–rectal bleeding
31	IV−7	F	–	57	–	–		
32	IV−8	M	–	57	–	–		
33	**IV−9**	F	–	Died	42	>100	Adenocarcinoma	Abdominal pain–lower track GI bleeding–constipation–cough
34	IV−10	M	–	53	–	–		
35	**IV−11**	F	DEL	40	39	>100	Tubular adenomatous polyposis with low‐grade dysplasia	Hemorrhoid–lower track GI bleeding–constipation
36	IV−12	F	–	58	–	–		
37	IV−13	F	–	54	–	–		
38	IV−14	F	–	52	–	–		
39	IV−15	F	–	46	–	–		
40	IV−16	M	WT	41	–	–		
41	IV−17	M	WT	38	–	–		
42	IV−18	M	WT	37	–	–		
43	IV−19	M	WT	34	–	–		
44	IV−20	F	WT	28	–	–		
45	IV−21	M	WT	27	–	–		
46	IV−22	F	WT	24	–	–		
47	V−1	M	–	1	–	–		
48	V−2	M	–	10	–	–		
49	V−3	F	WT	22	–	–		
50	V−4	M	WT	22	–	–		
51	**V−5**	F	DEL	28	24	>100	Multiple adenomatous polyps consistent with FAP	Abdominal pain–lower track GI bleeding–Melena
52	V−6	M	WT	26	–	–		
53	V−7	M	–	23	–	–		
54	V−8	F	–	Died	–	–		
55	V−9	M	WT	19	–	–		
56	**V−10**	M	DEL	17	16	>100	Small mucosal adenomatous polyps compatible with FAP	Constipation

Proband (IV‐11) was a 40‐year‐old symptomatic female diagnosed with FAP having unspecified clinical manifestations of gastrointestinal (GI) disorders such as constipation, lower GI bleeding due to hemorrhoid. She was a candidate for colonoscopy. There were more than one hundred polyps in the colonoscopy. She had colectomy, and pathological results demonstrated tubular adenomatous polyposis with low‐grade dysplasia.

### 3.2 Candidate mutation characterization

Proband (IV‐11) was screened for a panel of 14 genes (*APC, AXIN2, EPCAM, MLH1, MLH3, MSH2, MSH6, MUTYH, PMS1, PMS2, STK11, PTEN, SMAD4, and BMPR1A*) related with CRC by targeted next‐generation sequencing (NGS). A novel heterozygous large deletion, c.(135+1_136–1)_(*2113+1_*2114–1) spanning exon 3 to 16 [EX 3_16 DEL] with GenBank Accession: BankIt2072931 Seq1 MG712911 in *APC* gene [NCBI Reference sequence NM_000038], was identified in the proband (IV‐11).

### 3.3 Verification of the novel large deletion

**Figure 4:**
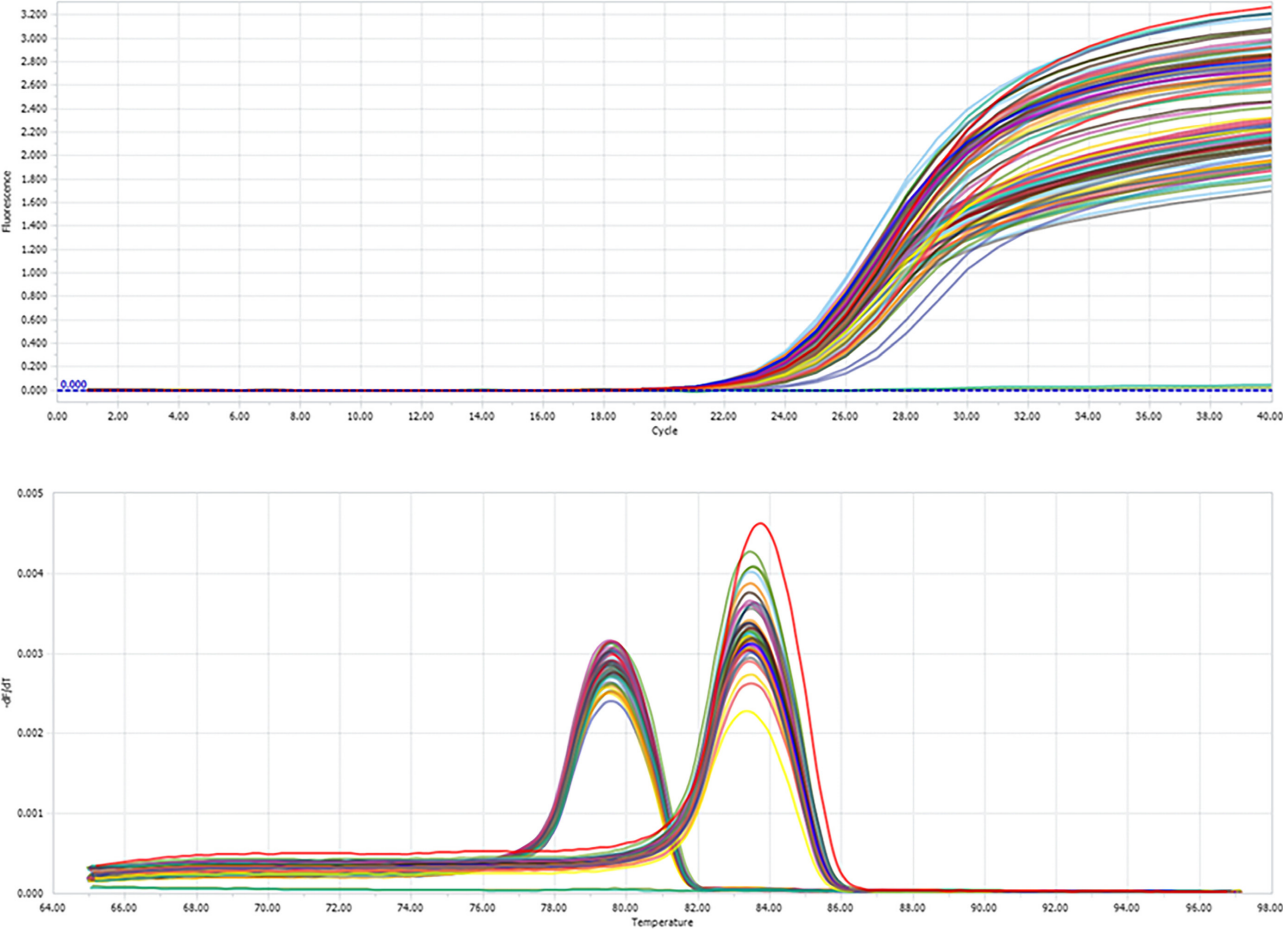
Quantitative real‐time PCR (qPCR) results. (a) Amplification curves. (b) Melting peaks. Exon 1 (at 83.5°C), Exon 14 (at 79.5°C)

As this is a large deletion mutation, for validation of the result, qPCR was undertaken, in the proband (IV‐11), in all affected family members (III‐5, III‐9, IV‐2, V‐5, V‐11), in 14 unaffected family members, and in 3 normal controls. Amplification curve and melting peak of all samples are shown in Figure 4.

The quantification of *APC* exon 14 was normalized to those of *APC* exon 1. For exon 14, 3 normal negative controls and 14 unaffected family members gave an approximately amplification level of 1.0, while the relative amplification from the proband (IV‐11) and all the affected family members (III‐5, III‐9, IV‐2, IV‐11, V‐5, V‐11) was approximately 0.5. There was a noticeable amplification level difference between affected and unaffected family members and normal controls, suggesting that the proband (IV‐11) and all the affected family members (III‐5, III‐9, IV‐2, V‐5, V‐11) have a novel heterozygous deletion of exon 3–16 of the *APC* gene that is associated with FAP in this family.

## 4 DISCUSSION

In our investigation, we identified a novel heterozygous large deletion, spanning exon 3 to 16 in *APC* gene [NCBI Reference sequence NM_000038] in all the affected family members of a five‐generation Iranian family with FAP. This heterozygous novel large deletion of *APC* gene is not present in ExAC database. In the current study, this novel heterozygous large germ line deletion exhibits the classical FAP phenotype with more than 100 polyps in colon. In this five‐generation Iranian family, clinical diagnosis of FAP has been carried out based on the endoscopic, clinical, and histopathological findings as well as their family history. The presence of polyposis and the autosomal dominant mode of inheritance in this family allowed us to diagnose it as a FAP disease. The diagnosis was confirmed by germ line mutation in *APC*.

**Figure 5:**
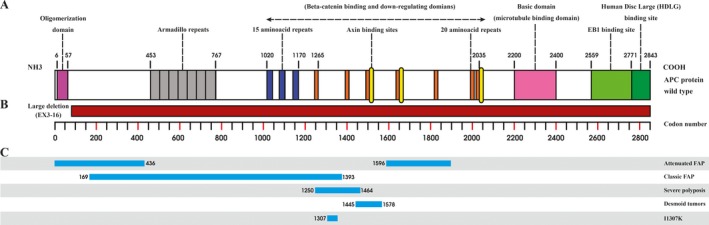
APC gene and protein. (a) Schematic diagram of the APC protein structure with functional domains, (b) large exon deletion (EX3– 16) on APC gene, (c) genotype–phenotype correlations on the APC gene

APC protein is a multi‐domain protein that contains binding sites for numerous proteins, as shown in Figure 5. The APC protein facilitates phosphorylation of beta‐catenin, targeting beta‐catenin for ubiquitination and degradation. In the absence of the APC protein, beta‐catenin accumulates in the nucleus and interacts with factors that up‐regulate the transcription of genes involved in cell cycle entry and progression (Aoki & Taketo, 2007). This novel heterozygous large deletion may lead to a truncated APC protein with loss of function in the APC/Axin/GSK‐3β destruction complex by complete absence of armadillo repeat region, β‐catenin binding site, DNA binding domain, microtubule‐binding site, EBI domain, and HDLG binding site.

Approximately 332 germ line mutations have been reported in *APC* gene (see Human Gene Mutation Database and references therein), responsible for the occurrence of FAP phenotype.

To date, 78 gross type deletions have been described in FAP patients (Zhang et al., 2016). De Rosa et al. (1999) described three unrelated kindreds, affected by FAP, with 5q submicroscopic deletions that encompass the entire gene and Cao, Eu, Seow‐Choen, Zao, & Cheah (2000) identified whole exon 11 and 14 deletions in the *APC* gene in three FAP families from Singapore. Aretz et al. (2005) also identified 14 different deletions in 26 patients, ranging from single exon to the whole gene including the promoter region. *APC* gene was examined by multiplex ligation‐dependent probe amplification (MLPA) assay in 14 FAP families. Large fragment deletions of exon 11 and 10A in one patient and a large fragment deletion of exon 15 start in another patient were detected by Sheng et al. (2010). In another study, Yamaguchi et al. (2014) identified germ line deletion of chromosome 5q22.1 – 22.2 (1.7 Mbp) in a patient with sparse type of FAP. In affected family members of one kindred with classical FAP, Lin et al. (2015) also identified a novel ~11 kb deletion localized 44 kb upstream of the transcription start site of *APC* that encompasses the *APC* 1B promoter and its exon. Besides, Zhang et al. (2016) reported a heterozygous large deletion in *APC* gene in a five‐generation Chinese family with FAP. Quadri et al. (2015) also characterized 6 deletions identified by MLPA method (three intragenic and three larger deletions encompassing the *APC* locus). Over the past decades, several studies have attempted to correlate specific *APC* mutations with clinical manifestations. Identification of the location of an *APC* mutation can predict the severity of the disease. It is highly possible that this phenotype–genotype correlation will open new perspectives to translational medicine, helpful in making therapeutic decisions (Friedl et al., 2001; Soravia et al., 1998; Wallis et al., 1994). Aretz et al. (2005) described a genotype–phenotype correlation since almost all deletions were detected in the 46 patients with predominant classical FAP, whereas no deletion was found in 93 patients with attenuated FAP. Marabelli et al. (2017) showed that promoter 1B deletion carriers from three Italian families all manifested a “classical” polyposis phenotype. Large *APC* deletions encompassing exon 14 have been found by Sieber et al. (2002) in patients with classical polyposis, and not in AFAP patients. By using MLPA method, Michils et al. (2005) showed that none of the 28 AFAP patients had a large deletion, while 15% of the mutation‐negative patients with classical polyposis had a genomic deletion.

Various studies did not detect large germ line *APC* deletions in attenuated polyposis patients; however, Pilarski, Brothman, Benn, & Shulman (1999) reported an attenuated polyposis case associated with the germ line deletion of the entire gene. Su, Kohlmann, Ward, & Lynch (2002) characterized one proband with the complete *APC* exon 15 germ line deletion, who had a phenotype consistent with AFAP. Nielsen *et al*. also identified *APC* deletions in 19 polyposis patients, who had negative *MUTYH* or *APC* point mutations. Eighty‐three percent of the families with germ line deletion displayed a CFAP phenotype. In their study, one family had a deletion expanding exons 1–5 and two other families carrying deletions encompassing exons 7–13 showing AFAP phenotype (Nielsen et al., 2007). Cattaneo et al. (2007) reported one patient carry an *APC* whole‐gene deletion with 100 polyps without any clinical manifestations. These observations elucidate that *APC* haploinsufficiency due to allelic deletion can occasionally create an attenuated/mild phenotype (Cattaneo et al., 2007).

FAP patients inherit one germ line mutation and in accordance to Knudson's two‐hit hypothesis, a second hit is required before adenomas can develop. The type of germ line mutation appears to determine the nature of the second hit to *APC* (Lamlum et al., 1999). Since this heterozygous novel deletion [EX3_16 DEL] is a large mutation removing almost the entire *APC* gene, the future work could investigate the second hit in this kindred. Understanding the molecular abnormalities in familial cases could help to identify some of the molecular mechanisms involved in sporadic CRC.

In conclusion, the current study describes a heterozygous novel gross deletion mutation, c.(135+1_136–1)_(*2113+1_*2114–1) with GenBank Accession: BankIt2072931 Seq1 MG712911 in *APC* gene in a five‐generation Iranian family with FAP. Our study expands germ line mutation spectrum of this gene in the Iranians' population. This novel finding contributes to a more comprehensive database of germ line mutation of the *APC* gene that could be used for molecular diagnosis of high‐risk mutation carriers and susceptibility of the disease for FAP patients.

## CONFLICT OF INTEREST

The authors declare no conflict of interest.
